# Feasibility, use and benefits of patient-reported outcome measures in palliative care units: a multicentre observational study

**DOI:** 10.1186/s12904-022-01123-y

**Published:** 2023-01-14

**Authors:** Evelyn Müller, Regine Mayer-Steinacker, Deniz Gencer, Jens Keßler, Bernd Alt-Epping, Stefan Schönsteiner, Helga Jäger, Bettina Couné, Luise Elster, Muhammet Keser, Julia Rauser, Susanne Marquardt, Gerhild Becker

**Affiliations:** 1 Department of Palliative Medicine, Medical Center – University of Freiburg, Faculty of Medicine, University of Freiburg, Robert-Koch Str. 3, 79106 Freiburg, Germany; 2 Department of Hematology and Oncology, Comprehensive Cancer Center, University Medical Center Ulm, Albert-Einstein-Allee 23, 89081 Ulm, Germany; 3 Department of Hematology and Oncology, Mannheim Cancer Center, Mannheim University Hospital, Mannheim Faculty of Medicine, University of Heidelberg, Theodor-Kutzer-Ufer 1-3, 68167 Mannheim, Germany; 4 Department of Anaesthesiology, Devision of Pain Medicine, University Hospital Heidelberg, Im Neuenheimer Feld 131, 69120 Heidelberg, Germany; 5 Competence Centre Palliative Care of Baden-Wuerttemberg, Baden-Wuerttemberg, Robert-Koch Str. 3, 79106 Freiburg, Germany; 6 Department of Palliative Medicine, University Hospital Heidelberg, Im Neuenheimer Feld 305, Heidelberg, 69120 Germany

**Keywords:** Palliative care, Palliative medicine, Outcome assessment, Patient-reported outcome measures

## Abstract

**Background:**

Research has shown that routinely assessed, patient-reported outcome measures (PROMs) have positive effects in patients with advanced oncologic diseases. However, the transferability of these results to specialist palliative care is uncertain because patients are more impaired and staff doubt the feasibility and benefits. The aim of this study is to evaluate the feasibility of patient self-assessment of PROMs, their use by staff and the benefits in palliative care wards.

**Method:**

A multicentre observational study was conducted in the context of the implementation of the Integrated Patient Outcome Scale (IPOS) in three specialist palliative care wards at university hospitals in Germany. All admitted patients who screened positive regarding their ability to complete questionnaires were asked to participate and complete the IPOS on paper weekly, with assistance if necessary. Feasibility of questionnaire completion (e.g. proportion of patients able to complete them), use (e.g. involvement of different professional groups) and benefit (e.g. unexpected information in IPOS as rated by treating physicians) were assessed. Staff members’ opinion was obtained in a written, anonymous evaluation survey, patients’ opinion in a short written evaluation.

**Results:**

A total of 557 patients were screened for eligibility, 235 were assessed as able to complete the IPOS (42.2%) and 137 participated in the study (24.6%). A majority needed support in completing the IPOS; 40 staff members and 73 patients completed the evaluation.

Unexpected information was marked by physicians in 95 of the 137 patient questionnaires (69.3%). The staff differed in their opinions on the question of whether this also improved treatment. A majority of 32 staff members (80.0%) were in favour of continuing the use of IPOS (4 against continuation, 4 no answer); 43 (58.9%) patients rated their overall experience of IPOS use as ‘positive’, 29 (39.7%) as ‘neutral’ and 1 (1.4%) as ‘negative’.

**Conclusions:**

While most staff wished to continue using IPOS, it was a challenge to integrate the effort to support the completion of IPOS into daily practice. Digital implementation was not successful, despite various attempts. To explore the effects on care and patient outcomes, multicentre cluster-randomised trials could be employed.

**Trial registration:**

German Clinical Trials Register DRKS-ID: DRKS00016681 (24/04/2019).

**Supplementary Information:**

The online version contains supplementary material available at 10.1186/s12904-022-01123-y.

## Background

The use of patient-reported outcome measures (PROMs) has become standard in research and clinical practice [1]. PROMs have also been introduced in palliative care: in 2016, the European Association for Palliative Care published a White Paper on outcome measurement in palliative care promoting the use of PROMs [2] and implementation projects promote their wide use, e.g. in Great Britain and Australia [3, 4].

Positive effects of PROMs on clinical routine are assumed to include identification of unmet patient needs, feedback on treatment effects, improved communication between patient/family and professionals, and quality management [5–7]. Studies have shown a positive influence of the routine assessment of PROMs on care processes and, in some cases, also on outcomes in palliative care patients [7–11].

It should be noted, however, that these studies were almost exclusively conducted in oncology settings, and data on the effects on inpatient specialist palliative care are scarce [6, 7, 11–13]. The results of studies in oncology should not be automatically transferred to palliative care settings. In specialist palliative care, patients are severely impaired and mostly in their last phase of life. Staffing ratios are higher, allowing for closer communication and support of patients. Furthermore, the positive impact of PROMs in oncology is partly due to the monitoring of potentially serious side effects of treatment [8, 10, 11, 14]. In specialist palliative care, chemo−/radio- or immunotherapy is rarely performed [15].

Recognising the fact that, sooner or later, self-assessment becomes impossible in the last phase of life [13], many common questionnaires used in palliative care and oncology offer proxy versions [16, 17]. Etkind et al. proposed the term “patient-centred outcome measures” (PCOMs) to reflect the need and value of both patient-reported and proxy-reported measures, while emphasising the perspective of the patients in palliative care [6].

In addition to the challenges with regard to patients, doubts about the feasibility and benefits are common among staff working in specialist palliative care contexts [6, 7, 18–20]. There is a range of institutional barriers [6, 20]. Interviews with palliative care staff indicate that many of them are convinced that their competence, experience, intuition, and inter-professional communication leave little room for improvement in terms of identification of patients’ needs. They fear loss of personal contact with patients and do not believe that PROMs can adequately reflect the complexity of their patients’ needs [19]. The assumption that filling in the questionnaires adds another burden to already burdened patients often results in ‘gatekeeping’ [19, 21, 22].

Implementation of PROMs requires a long-term strategy, time and human resources as well as the continuous commitment and attention of leaders and staff [6, 18]. It is unlikely to be successful if staff are not convinced of its benefit and feasibility.

Against this background, we attempted to implement structures and processes for clinical use of self-assessed PROMs in three different palliative care units. This included, where possible, digital means of capturing and documenting PROMs. Digital documentation of an assessment is a prerequisite for cost accounting of inpatient palliative treatment in Germany. The implementation of PROMs was primarily for study purpose. Continuing the use of PROMs after the project was a local decision.

The aim of the project was to collect data on effort and benefit of PROM use in specialist palliative care wards. In particular, we collected data on feasibility of PROM completion by patients, the processes of use by staff, the extent of information on patient distress in PROMs that is unexpected to the treating physician and how staff perceived the benefits of PROM use e.g. for the treatment.

## Methods

### Study design

We conducted a multicentre observational study of the implementation of PROMs in palliative care units. The study was approved by the ethics committees of all participating institutions (details: *Ethics and consent* section).

### Setting

The project was conducted in three palliative care units (Freiburg, Mannheim, Ulm) located at different university medical centres in the federal state of Baden-Wuerttemberg, southwest Germany. These are part of the Competence Centre Palliative Care (KompetenzZentrum Palliative Care), a network of the palliative care units at the university hospital centres in Baden-Wuerttemberg. Two of the units are closely linked to oncology wards, one is independent of structural links. The wards have 10–14 beds and cared for 155–335 patients in 2019. Periods of implementation and patient recruitment varied but were all within the period from April 2019 to June 2020.

### Implementation strategy

In a workshop with representatives of the participating palliative care units, study objectives, implementation strategy, the selection of a common questionnaire, measurement frequency and measurement points were discussed and agreed upon. Measures for implementation were derived from relevant literature [6, 7, 20]:

Local steering groups including executives and team members were formed and regularly discussed, consented and reviewed planning and progress, employing the PDCA cycle [23] (PDCA: *p*lanning, implementing [*d*oing], *c*hecking and, if necessary, changing processes [*a*cting]). The steering groups received standardised forms in which they were asked to document e.g. targets of PROM use in their ward, local workflows and responsibilities for patient information, PROM collection and PROM review by staff—including the rationale and records of changes over time. Each ward had a study assistant who was trained in study conduct and the use of PROMs, and had the role of a local expert who passes on their knowledge. Study assistants were also a human resource for reliable collection of the PROMs. The plan was for them to initiate the collection of PROMs and oversee the process in the first weeks before handing it over step by step to the teams. Furthermore, the Freiburg study team visited all wards to provide information on PROM use and study conduct and the external study assistants were trained at the ongoing project at the Freiburg site before starting at their site.

### Screening and patient recruitment

Physicians consecutively screened all newly admitted adult patients to determine whether they were physically and cognitively able to participate in the project and had sufficient German language skills to communicate their needs and symptoms themselves. The screening was based on the professional experience and expertise of the physicians taking the medical history on admission to the ward. Criteria were e.g. the patient’s verbal (statements of inability to concentrate / comprehend / listen / answer, conclusiveness of statements) and non-verbal responses (reduced vigilance, disorientation). Inability to read or write was not a reason for exclusion of patients if assisted completion of PROMs was possible. Patients fulfilling these criteria were informed about the project and written informed consent was obtained if they were willing to participate. In cases of continued care by the in-house specialist outpatient palliative care teams after discharge or on readmission to hospital, patients and staff were free to continue using PROM, but this was not counted as a second study participation.

### Administration and use of PROMs

An extended form of the Integrated Patient Outcome Scale (IPOS [24]) was administered at admission, weekly and at discharge to all study patients. When patients were discharged, PROM use did not continue unless they were receiving further care through directly associated outpatient palliative care. The IPOS is a 10-item holistic, valid and reliable multidimensional assessment of needs and symptoms for patients with advanced, severe diseases. We added eight dichotomous items (yes/no) concerning practical problems (e.g. housing situation, insurance, work/school, transport, childcare, financial worries, care for others, other) that were of special interest to social workers. Patients received paper-based questionnaires from members of staff or study assistants. If study assistants or staff felt patients might need help completing PROMs, they offered support. Support could be given by a staff member/the study assistant or relatives. The implementation of the support could vary according to the situation and patient needs, e.g. explanation of terms, reading out the questions or a more informal conversation. The specific situation in which PROM completion occurred was determined locally (e.g. during anamnesis or special appointment). The completed questionnaires were collected and given to the treating physician. Further procedures for passing the questionnaires on to other staff members (e.g. inclusion in medical records or scanning for sending by email) or discussing them within the team depended on local decisions in the steering group.

### Evaluation

A written evaluation survey for palliative care staff was conducted in the final weeks of the study. This covered the use and benefits of PROMs and the desire to continue/discontinue their use, predominantly documented through closed questions. A description of the development and pre-testing, as well as a full version of the questionnaire, are included in Additional file 1. The staff at the palliative care units were asked to complete the evaluation questionnaire if they were in any way involved in the implementation and/or use of PROMs (inclusion criteria). This included either active (e.g. assessment or review of PROMs) or passive involvement (e.g. attending team meetings where PROMs were discussed). The survey was anonymous and questionnaires were returned in closed envelopes within the respective institutions or mailed to the study group in Freiburg. In order to ensure anonymity in the small sample, gender, age and professional experience were not collected.

Patients were asked to complete a short evaluation questionnaire with closed and open-ended questions (with or without assistance) after completing at least the second PROM. This was to ensure that they had experience not only of completing PROMs but also of staff using them. A description of the questionnaire and all results are included in Additional file 3*.*

### Data collection

The following data were documented:

*Feasibility of PROM completion:* The proportion of patients able to complete PROMs (independently or assisted) was assessed in a two-stage procedure. Firstly, the treating physicians assessed patients’ cognitive and physical abilities and German language skills during screening at admission, which includes taking a medical history with patients (and/or relatives). Secondly, study assistants re-evaluated the same parameters on their first visit, usually 1–3 days after admission, which was necessary due to the sometimes rapidly declining health of palliative care patients. The study assistants all had a professional medical background and were experienced in conversation situations with cancer and palliative care patients. If both physician and study assistant assessments were positive, it was assumed that patients were able to complete PROMs.

Furthermore, for non-participants, the reasons for refusal were requested. For participants, the proportion of patients able and willing to complete the questionnaire without support was determined.

*Benefit and use:* Physicians marked unexpected information in the first completed patient questionnaire (admission) with a highlighter. To ensure that this was done, assistants stuck a Post-It Note on the questionnaires that read “Please mark the information on this questionnaire that you would not have expected” (translated from German). The evaluation also included various items concerning both benefit and use. An English translation of the questionnaire is provided in Additional file 1.

### Analysis

The study aimed at a descriptive and explorative analysis for all study questions. Missing data are reported as we do not expect them to be random (no imputation). Open-ended questions in evaluations were categorised by a member of the study team by content and two other members checked if their understanding of categories and the allocation of answers were comprehensible (translations of all original answers and categories are reported in Additional files 1 and 3). The following associations were explored with inferential analysis: (a) The association between distress levels and information being unexpected as well as differences between professional groups in the evaluation survey were investigated by Chi^2^-test (two-sided). (b) Kendall’s Tau was employed to explore the correlation between the use of PROMs and evaluation of their benefits as well as the wish to continue their use. Due to the explorative nature of the study, alpha adjustment was not applied; alpha level was set at 5% (2-sided).

## Results

### Samples

**Fig. 1:**
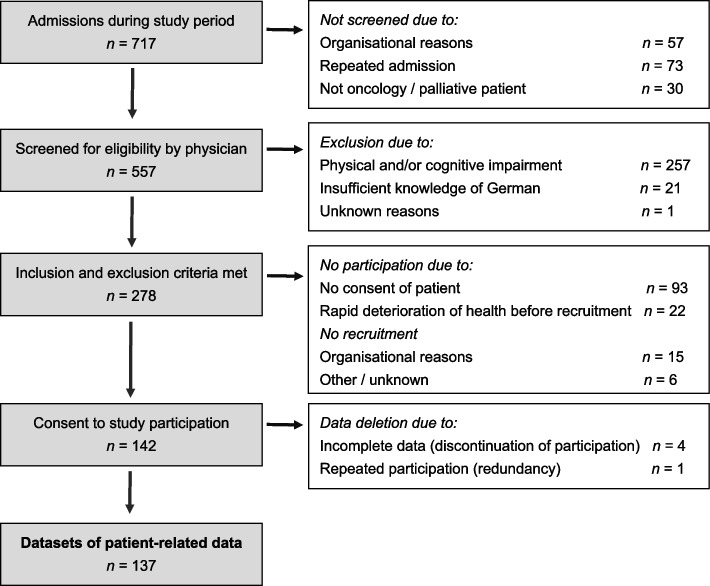
Flowchart of screening and recruitment

The implementation periods differed between the palliative care units and were 8, 10 and 13 months in 2019/20. Implementation and data collection ended abruptly and early due to the Covid-19 pandemic. For a full account on the screening and recruitment process of **patients**, see Fig. 1. Of the 557 patients that were screened for eligibility at admission by the physicians, 278 met the inclusion criteria. Data from 137 patients were analysed. Table 1 shows the characteristics of the patient sample.

**Table 1:** Characteristics of participating patients (*n* = 137)

**Age**	**m (sd)**	**min–max**
	63.9 (14.3)	27–90
**Gender**	***n***	**%**
Female	66	48.2
Male	71	51.8
**Living situation**	***n***	**%**
Living alone	45	32.8
Living with others	92	67.2
**General condition – ECOG**	***n***	**%**
0 – fully active	1	0.7
1 – restricted in strenuous activity, light work possible	10	7.3
2 – ambulatory, capable of self-care, unable to work	33	24.1
3 – capable of only limited self-care, 50% confined to bed / chair	57	41.6
4 – completely disabled, no self-care, confined to bed / chair	16	11.7
5 – dead	0	0.0
Missing data	20	14.6
**Main diagnosis**	***n***	**%**
*Neoplasm of …*		
Digestive organs	24	17.5
Respiratory/intrathoracic organs	21	15.3
Female genital organs	19	13.9
Male genital organs	17	12.4
Other (< 10% of sample per diagnosis)	52	37.9
*Non-oncologic*		
COPD (2x), ALS, heart failure	4	2.9
**Reason for end of PROM-collection**	***n***	**%**
Regular discharge	92	67.2
Death	15	10.9
Deteriorated health	21	15.3
Drop-out – patient decision	3	2.2
Drop-out – unknown reason *or* Covid-19	6	4.4

Abbreviations: *m* Mean value, *sd* Standard deviation, *min* Minimum, *max* Maximum, *n* Number of cases, *ECOG* Eastern Cooperative Oncology Group (ECOG) performance status [25], *COPD* Chronic Obstructive Pulmonary Disease, *ALS* Amyotrophic lateral sclerosis, *Covid-19* Corona virus disease 19

In the **evaluation**, 12 physicians, 15 nurses, 9 staff members with psychosocial professions and 4 indicating *other profession* participated (*n* = 40). The number of questionnaires distributed was not documented by one palliative care unit; therefore, no overall response rate can be given. In the two centres with controlled distribution 36 questionnaires were distributed and 34 were returned (94% response rate). In the centre without controlled distribution 6 members of staff returned the questionnaires.

### Feasibility of PROM completion

235 (42.2%) of 557 patients were assessed as being able to complete PROMs by the physicians at admission and during re-evaluation by the study assistants; 257 (46.1%) were assessed as physically and/or cognitively too impaired for self-assessment by physicians, 22 (3.9%) patients had rapidly deteriorating health prior to recruitment, 21 (3.8%) had insufficient knowledge of the German language and 22 were not sufficiently assessed regarding the matter (3.9%).

It is important to note that the rate of patients assessed as able to complete PROMs differed widely between the three palliative care units, with proportions being similar for two of them (37.5 and 38.6%) and the third being much higher (63.6%). Health care data from these 3 units (all for 2019) indicate variation in patients’ health status as a likely explanation. The palliative care unit with the far higher rate of eligible patients had the lowest proportion of patients dying on the ward with 36% (vs. 42 and 50%) and the longest average length of stay of 14 days (vs. 12 and 11 days) in 2019. Recruitment took place for 235 patients—142 consented to participate, 93 patients did not consent. When asked if the reason for non-participation could be documented, 55 named physical and / or psychological distress (*n* = 12 non-health / burden-related reasons, *n* = 26 not specified).

From the 142 patients that consented to participate, 137 data sets were available for analysis. The majority of participants (*n* = 101; 73.7%) received support in completing the IPOS, 36 (26.3%) completed it by themselves (for detailed analyses see Additional file 2).

### Use by staff

**Fig. 2:**
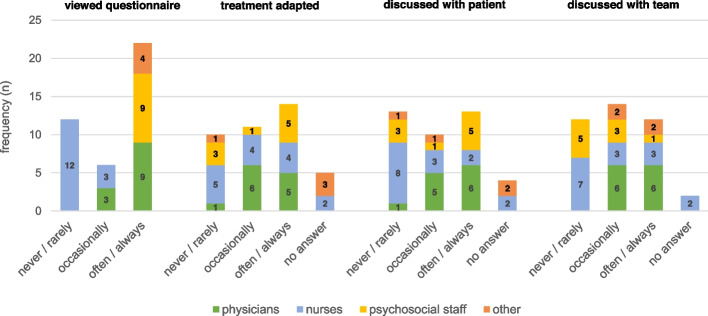
Use of PROMs by staff members as reported in evaluation survey (*n* = 40)

Physicians regularly viewed patients’ questionnaires (see Fig. 2), as determined by the study design. Psychologists and social workers also showed interest, e.g. in one ward they were sent copies by email. By contrast, nurses rarely viewed the questionnaires (significant differences between professions, Chi^2^ (12) = 43.7, *p* < .001); however, they were involved in one ward as the PROM results were regularly discussed in daily inter-professional case reviews. There were no significant differences between professional groups in reports on the use of PROMs for the adaption of treatment (Chi^2^ (12) = 15.9, *p* = .19) and in discussions with patients (Chi^2^ (12) = 14.3, *p* = .29) and the team (Chi^2^ (12) = 19.3, *p* = .08). Overall, about a third of the responding staff members indicated only rare involvement. Involvement in PROM use (viewing the questionnaires, discussing them within the team or with patients, adapting treatment) generally correlated significantly with more positive ratings of benefit. The only aspect of use that showed a significant correlation with the wish to continue PROM use was the experience that PROM use resulted in an adaption of treatment (see Additional file 1, Table S2 for all correlations). The effort of PROM use was rated as *rather low* by 12 members of staff, 10 rated it as *rather high* and 1 as *very high* (*n* = 17 *could not say*; original item in Additional file 1, Table S1, Item 5).

### Benefit

Of the 137 patient questionnaires reviewed, physicians marked unexpected information (anywhere, including open-ended questions) in 95 (69.3%). Tables 2 and 3 give an overview of the frequency and level of distress indicated for the symptoms and problems covered in the IPOS in the sample. Furthermore, the frequency of unexpected information being marked and its association with the level of distress are included.

**Table 2:** Distress levels and marking of unexpected information in IPOS: physical symptoms

Reported distress		Patient report (IPOS) ***n*** (% column)	Marked ***unexpected*** ***n*** (% row)	Association distress level & marking ***unexpected*** Chi^2^-test (two-sided)*
**Pain** **(*n*** **= 137)**	not at all/slight**	49 (35.8)	3 (6.1)	Chi^2^ (4) = 17.1 *p* = .002
	moderate	34 (24.8)	5 (14.7)	
	severe/overwhelming	54 (39.4)	13 (24.1)	
	overall	137 (100.0)	21 (15.3)	
**Shortness of breath** **(*n*** **= 136)**	not at all/slight	88 (64.7)	3 (3.4)	Chi^2^ (4) = 17.1 *p* = .002
	moderate	19 (13.9)	4 (21.1)	
	severe/overwhelming	29 (21.2)	6 (20.7)	
	overall	136 (100.0)	13 (9.6)	
**Weakness or lack of energy** **(*n*** **= 136)**	not at all/slight	20 (14.7)	1 (5.0%)	Chi^2^ (4) = 2.8 *p* = .588
	moderate	39 (28.7)	2 (5.1%)	
	severe/overwhelming	77 (56.6)	7 (9.1%)	
	overall	136 (100.0)	10 (7.4)	
**Nausea** **(*n*** **= 136)**	not at all/slight	93 (68.4)	1 (1.1)	Chi^2^ (4) = 24.3 *p* < .001
	moderate	13 (9.6)	1 (7.7)	
	severe/overwhelmingly	30 (22.1)	7 (23.3)	
	overall	136 (100.0)	9 (6.6)	
**Vomiting** **(*n*** **= 136)**	not at all/slight	104 (76.5)	1 (1)	Chi^2^ (4) = 27.8 *p* < .001
	moderate	9 (6.6)	0 (0.0)	
	severe/overwhelming	23 (16.9)	6 (26.1)	
	overall	136 (100.0)	7 (5.1)	
**Poor appetite** **(*n*** **= 136)**	not at all/slight	68 (50.0)	4 (5.9)	Chi^2^ (4) = 1.7 *p* = .781
	moderate	20 (14.7)	2 (10.0)	
	severe/overwhelming	48 (35.3)	3 (6.3)	
	overall	136 (100.0)	9 (6.6)	
**Constipation** **(*n*** **= 135)**	not at all/slight	81 (60.0)	1 (1.2)	Chi^2^ (4) = 25.3 *p* < .001
	moderate	15 (11.1)	3 (20.0)	
	severe/overwhelming	39 (28.9)	10 (25.6)	
	overall	135 (100.0)	14 (10.4)	
**Sore or dry mouth** **(*n*** **= 136)**	not at all/slight	49 (36.0)	0 (0.0)	Chi^2^ (4) = 16.8 *p* = .002
	moderate	33 (24.3)	6 (18.2)	
	severe/overwhelming	54 (39.7)	16 (29.6)	
	overall	136 (100.0)	22 (16.2)	
**Drowsiness** **(*n*** **= 137)**	not at all/slight	49 (35.8)	2 (4.1)	Chi^2^ (4) = 6.2 *p* = .182
	moderate	40 (29.2)	3 (7.5)	
	severe/overwhelming	48 (35.0)	8 (16.7)	
	overall	137 (100.0)	13 (9.5)	
**Poor mobility** **(*n*** **= 137)**	not at all/slight	22 (16.1)	1 (4.5)	Chi^2^ (4) = 1.6 *p* = .806
	moderate	27 (19.7)	1 (3.7)	
	severe/overwhelming	88 (64.3)	8 (9.1)	
	overall	137 (100.0)	10 (7.3)	

* The Chi^2^-test was calculated for the five-point Likert scale without data reduction to the three-point format shown here; this is solely for the sake of clearer presentation

** For a more concise presentation of some results, levels of distress of specific symptoms are not presented as the original five-point scale but reduced to three categories

**Table 3:** Distress levels, marking of unexpected information in IPOS: psychosocial and practical problems

Reported distress		Patient report (IPOS) ***n*** (% column)	Marked ***unexpected*** ***n*** (% row)	Association distress level & marking unexpected Chi^2^-test (two-sided)*
**Anxious/worried about illness/treatment** **(*n*** **= 136)**	not at all/occasionally**	24 (17.6)	6 (25.0)	Chi^2^ (4) = 21.2 *p* < .001
	sometimes	46 (33.8)	1 (2.2)	
	most of the time/always	66 (48.5)	15 (22.7)	
	overall	136 (100.0)	22 (16.2)	
**Family/friends anxious/worried about you** **(*n*** **= 134)**	not at all/occasionally	4 (2.9)	1 (25.0)	Chi^2^ (4) = 9.1 *p* = .027
	sometimes	24 (17.6)	0 (0.0)	
	most of the time/always	106 (77.9)	5 (4.7)	
	overall	134 (100.0)	6 (4.5)	
**Feeling depressed** **(*n*** **= 134)**	not at all/occasionally	40 (29.4)	3 (7.5)	Chi^2^ (4) = 22.6 *p* < .001
	sometimes	51 (37.5)	0 (0.0)	
	most of the time/always	43 (31.6)	12 (27.9)	
	overall	134 (100.0)	15 (11.2)	
**Feeling at peace** **(*n*** **= 133)**	always/most of the time	90 (66.2)	3 (3.3)	Chi^2^ (4) = 10.6 *p* = .031
	sometimes	22 (16.2)	2 (9.1)	
	occasionally/not at all	21 (15.4)	4 (19.0)	
	overall	133 (100.0)	9 (6.8)	
**Can share feelings as much as you want** **(*n*** **= 134)**	always/most of the time	110 (80.9)	1 (0.9)	Chi^2^ (4) = 37.6 *p* < .001
	sometimes	10 (7.4)	1 (10.0)	
	occasionally/not at all	14 (10.3)	3 (21.4)	
	overall	134 (100.0)	5 (3.7)	
**As much information as wanted** **(*n*** **= 134)**	always/most of the time	113 (83.1)	2 (1.8)	Chi^2^ (4) = 40.4 *p* < .001
	sometimes	13 (9.6)	6 (46.2)	
	occasionally/not at all	8 (5.9)	1 (12.5)	
	overall	134 (100.0)	9 (6.7)	
**Practical problems addressed** **(*n*** **= 132)**	none or addressed/mostly addressed	96 (70.6)	2 (2.1)	Chi^2^ (4) = 17.1 *p* = .002
	partly addressed	24 (17.6)	0 (0.0)	
	hardly/not addressed	12 (8.8)	3 (25.0)	
	overall	132 (100.0)	5 (3.8)	

* The Chi^2^-test was calculated for the five-point Likert scale without data reduction to the three-point format shown here; this is solely for the sake of clearer presentation

** For a more concise presentation of some results, levels of distress of specific symptoms are not presented as the original five-point scale but reduced to three categories

The physical symptoms with the highest distress levels in the sample were weakness and reduced mobility (> 50% of patients indicated severe/overwhelming), followed by drowsiness, sore or dry mouth, loss of appetite and pain (> 35%). The symptoms most often marked as unexpected information by physicians were pain (15.3%) and sore or dry mouth (16.2%). For most symptoms, higher distress levels were significantly more likely to be unexpected.

With regard to psychosocial and practical problems (table 3), anxiousness and worries about illness and treatment experienced by patients and their relatives were the most frequent problems and also those most frequently marked as unexpected by physicians. Again, the level of distress was associated with unexpected information being marked by the physicians.

**Fig. 3:**
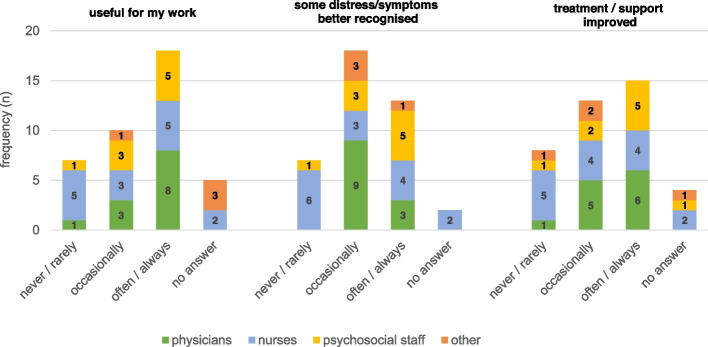
Assessment of usefulness of PROMs as reported in the evaluation survey (*n* = 40)

Of the 137 study participants, 73 (53.3%) completed evaluation (full results and questionnaire see Additional file 3). The majority (*n* = 43; 58.9%) rated their overall experience as ‘positive’, while 29 (39.7%) rated it as ‘neutral’ and 1 (1.4%) as ‘negative’. In their answers to the corresponding open-ended question, they described perceived benefits, e.g. that supported completion or PROM use in general helped in their reflection on and coping with the current situation or that the questionnaire was used as an additional means of communication with staff. However, other patients described PROM use as exhausting or annoying, or mentioned doubts about its benefits. In the staff evaluation survey, 18 of the 40 responding staff members (45.0%) regarded the use of PROMs as often or always useful for their work (see Fig. 3); another 10 respondents considered it as occasionally useful. The positive ratings were lower for more specific purposes of PROMs, e.g. improvement of support/treatment (*n* = 15 often/always) and recognition of distress (*n* = 13 often/always). There were no significant differences in the ratings between professions.

A majority of 32 of the 40 respondents (80.0%) were in favour of continuing PROM use (n = 4 no continuation, n = 4 no answer), even though in an overall benefit rating, only 11 participants rated the benefit as rather high or high (*n* = 12 *rather low*, *n* = 17 *can’t say*). However, 16 of the 32 in favour of continuation would like to see changes; most frequently a change to electronic data collection and documentation, but also changes in the communication processes concerning the questionnaire within the team and in the contents of the questionnaire. Reasons in favour of discontinuation (*n* = 4) included an inadequate cost–benefit ratio, fear of ‘even more paperwork’ and burdening the patients.

## Discussion

The question of PROM use is a question of effort and benefit on the patient, staff and institutional levels. Our study allowed staff to experience the benefits and draw their own conclusions. The conclusion of the members of staff was mainly positive. However, the data also demonstrate the limits of self-assessment and the necessary effort and barriers of implementation in specialist palliative care.

The reported informational benefit in everyday practice is supported by studies that have compared proxy- and self-assessments, showing high rates of noncongruence: Staff often underestimate the patients’ symptoms and distress [26–28], while informal caregivers often overestimate it [29, 30]. As to whether this information is useful, e.g. for self-reflection of patients, communication and treatment, a wide range of experiences and opinions were reported both among staff and patients, similar to other studies [31]. The correlation between participation in the use of PROM and positive evaluation of the benefits most likely illustrates a reciprocal influence: a positive attitude to PROMs implies a willingness to use them and the experience of their benefits leads to a reconsideration of doubts.

On the cost side, it must be taken into account that the collection of PROMs requires not only adequate cognitive and physical abilities of the patient but also considerable resources on the side of the staff. Both the high rates of patients being unable to complete questionnaires [31] and the high need of support have been confirmed by other studies [13]. Supporting patients in completing the questionnaires also turned out to be a challenge for staff, as this involves a balancing act between self-assessment and proxy-assessment [5].

So if the majority of staff would like to continue PROM use, why did two out of the three teams not even plan to do so directly after the project? Two barriers stand out in the experience of the project teams: The provision of support by team members during completion of PROMs and the lack of digital collection and documentation of PROMs.

PROM collection by staff is not only a question of effort and time, but also of integration into everyday practice. For example, staff found completing the PROM at the time of admission to be too demanding for patients. As study assistants were always available, attempts may have been terminated prematurely.

The introduction of IPOS at least in electronic patient documentation was the development most urgently requested by staff in the evaluation. It was planned originally that a partner company would realise digital implementation within the project. However, this company failed to implement the necessary data protection requirements. Further attempts within the project time frame also failed, e.g. due to skill shortages in alternative companies. Consequently, PROMs had to be collected in addition to the ongoing electronic proxy-assessment documentation required for cost accounting. The advantages of digital documentation (and if possible collection) of PROMs are not only in the use for accounting but also in the electronic support of the interpretation through graphic illustration of progression, alerts for patient entries with a high probability of need for action and the easy accessibility of results for all staff. Once electronic IPOS documentation is in place, it is likely to lead to everyday use of IPOS, but whether as self- and / or proxy-assessment remains to be seen.

### Limitations

Limitations of our study include a preliminary termination of the implementation process, as two of the three participating wards were closed down due to the Covid-19 pandemic. As the 12 month implementation phase was not fully completed there, results on feasibility at the institutional level stay preliminary. Attempts to fade out the support of study assistants and carry out data collection by staff were not successful in the long run in two out of the three palliative care units.

The feasibility of PROM completion by patients might have been underestimated in our project. Doubts about benefit and gatekeeping [19, 21, 22] might have affected the physicians’ assessments during screening, reducing the proportion of patients classified as able to complete PROMs. The problem might have been exacerbated by the fact that patient participation in the study also included the recruitment process for the study and evaluation of patients, a higher effort than only PROM use in everyday practice would require. This effort may also have affected the willingness of patients to participate. Furthermore, a similar effect might have occurred regarding the need for support in completion of PROMs—study assistants and staff might have been too accommodating, underestimating the patients’ abilities.

In this publication only results of evaluation surveys are reported. Results might be biased by question selection and question wording by the study team, socially desirable responses and also selective sampling. The results from the evaluation by patients are especially biased as the sample only includes patients that were willing and able to complete PROMs at least twice. Interviews with staff were conducted to complement and deepen the findings concerning the use of PROMs in palliative care units, and we plan to make these results available in a further publication.

### Strengths

This study provides data on the use of PROMs in specialist palliative care units. Even though the framework conditions vary between facilities and health systems and may lead to different levels of necessary effort or benefit, the challenges of PROM use in highly impaired patients are comparable. In this respect, the fundamental conclusions are nevertheless transferable to other specialist palliative care units in Germany and internationally.

## Conclusions

This study provides insight on feasibility, use and benefit of PROM-use in specialist palliative care. Our results confirm the apprehension that a relevant share of patients are too burdened to complete questionnaires without support. At the same time, the assumption of many staff members that the close contact and extensive experience of the team in specialist palliative care hardly allows for additional benefits from PROMs is not confirmed.

Self-assessment is appreciated by many palliative care team members after experiencing its benefits, despite the awareness of its limitations in specialist palliative care. The list of recommendations for successful implementation includes e.g. defined aims, processes and responsibilities, training, facilitators and digital implementation [5, 6, 17]. However, sustainable implementation also involves soft factors, especially convincing the team of the benefits and feasibility. To improve this, further measures should be considered, such as marking unexpected information and team discussion and evaluation, focusing not only on improvements and problems, but also on successes.

Without motivation and competence of staff, there is a risk that PROM use will degenerate into a duty to document instead of an opportunity for patients to express their needs.

Even though staff reported positive effects on care, this is an evaluation, an account of their perception and experience. To explore the effects on care and—more importantly—patient outcomes, multi-centre cluster-randomised trials would be necessary. This could provide strong evidence and justify widespread use in everyday practice in specialist palliative care.

## Supplementary Information

**Additional file 1.** Evaluation Survey.

**Additional file 2.** Statistics on staff support for patients’ self-assessment of PROMs by type of discharge and point of measurement.

**Additional file 3.** Patient evaluation.

## Data Availability

The datasets used and/or analysed during the current study are available from the corresponding author on reasonable request.

## Abbreviations

PROM: patient-reported outcome measure
PCOM: patient-centred outcome measure
IPOS: Integrated Patient Outcome Scale
