# Supplementary material for: Feasibility, use and benefits of patient-reported outcome measures in palliative care units: a multicentre observational study
Source: BMC Palliat Care. 2023 Jan 14;22:6. doi: 10.1186/s12904-022-01123-y (PMC9839955; doi:10.1186/s12904-022-01123-y)
Supplement: Supplementary file 1 — Additional file 1. Evaluation Survey. [file 12904_2022_1123_MOESM1_ESM.pdf]

Additional file 1 – Evaluation Survey

Development of Questionnaire:

The first version of the evaluation questionnaire was developed by a multi-professional study group (nursing, psychometrics, education, clinical psychology) based on the literature on effects and implementation of PROMs in palliative care [1–4]. The items were selected to reflect relevant aspects of the implementation process of PROMs, the benefits that were most relevant to evaluate on the basis of the often reported doubts of their usefulness in palliative care.

A pre-test was conducted by means of cognitive interviews with five individuals involved in the implementation of PROMs in the clinical setting (2 nurses, 2 doctors, 1 social worker). The pre-test examined the comprehensibility of question wording, the applicability of the scales used, the face validity of the contents and missing topics, as well as possibilities for shortening the questionnaire, from the users' point of view. The questionnaire was revised according to the results in an iterative process.

Table S1: Questionnaire for Evaluation Survey including results (n (%))

**1. Please mark the answer that fits best.**	never	rarely	occasionally	often	always	not answered
I reviewed the patients' statements in the questionnaire myself.	10 (25 %)	2 (5 %)	6 (15 %)	7 (17.5 %)	15 (37.5 %)	0 (0 %)
The information from the questionnaire was discussed by the team.	3 (7.5 %)	12 (30 %)	14 (35 %)	9 (22.5 %)	2 (5 %)	0 (0 %)
Some of the patients' needs were better recognised through the systematic assessment.	4 (10 %)	3 (7.5 %)	18 (45 %)	11 (27.5 %)	2 (5 %)	2 (5 %)
I have adapted treatment or support based on the patients' statements in the questionnaire.	5 (12.5 %)	5 (12.5 %)	11 (27.5 %)	8 (20 %)	6 (15 %)	5 (12.5 %)
I have used the information provided by the patients to address specific issues with the patients.	7 (17.5 %)	6 (15 %)	10 (25 %)	9 (22.5 %)	4 (10 %)	4 (10 %)
I have used the information provided by the patients to address the patients' burdens with the team or with colleagues.	5 (12.5 %)	7 (17.5 %)	14 (35 %)	10 (25 %)	2 (5 %)	2 (5 %)
The patients' statements in the questionnaire were useful for my work.	3 (7.5 %)	4 (10 %)	10 (25 %)	10 (25 %)	8 (20 %)	5 (12.5 %)
The treatment and support of patients has improved overall due to the systematic assessment of patient-reported distress.	3 (7.5 %)	5 (12.5 %)	13 (32.5 %)	12 (30 %)	3 (7.5 %)	4 (10 %)

**2. I would not have noticed some of the patients' distress without the questionnaire data.**					
agree	somewhat agree	somewhat disagree	disagree	I can't say	not answered
10 (25 %)	15 (37.5 %)	3 (7.5 %)	4 (10 %)	7 (17.5 %)	1 (2.5 %)

**3. Where has the systematic assessment and integration of patient-reported distress led to improvements? Where has something worsened?**									
	worsened						improved	I can't say	Not answered
	-3	-2	-1	0	1	2	3		
Treatment of physical distress/symptoms	0 (0 %)	0 (0 %)	0 (0 %)	8 (20 %)	7 (17.5 %)	8 (20 %)	1 (2.5 %)	15 (37.5 %)	1 (2.5 %)
Treatment of mental distress	0 (0 %)	0 (0 %)	1 (2.5 %)	3 (7.5 %)	15 (37.5 %)	9 (22.5 %)	0 (0 %)	11 (27.5 %)	1 (2.5 %)
Counselling for social problems	0 (0 %)	0 (0 %)	0 (0 %)	4 (10 %)	14 (35 %)	7 (17.5 %)	5 (12.5 %)	9 (22.5 %)	1 (2.5 %)
Support during existential crises	0 (0 %)	1 (2.5 %)	0 (0 %)	9 (22.5 %)	7 (17.5 %)	4 (10 %)	2 (5 %)	16 (40 %)	1 (2.5 %)
Support with spiritual/religious concerns	0 (0 %)	1 (2.5 %)	1 (2.5 %)	7 (17.5 %)	6 (15 %)	4 (10 %)	0 (0 %)	20 (50 %)	1 (2.5 %)
Practitioner–patient communication	0 (0 %)	0 (0 %)	0 (0 %)	5 (12.5 %)	11 (27.5 %)	8 (20 %)	3 (7.5 %)	11 (27.5 %)	2 (5 %)
Communication in the team about the patients' distress	0 (0 %)	0 (0 %)	0 (0 %)	6 (15 %)	15 (37.5 %)	8 (20 %)	0 (0 %)	10 (25 %)	1 (2.5 %)
Holistic support and treatment	0 (0 %)	0 (0 %)	0 (0 %)	6 (15 %)	11 (27.5 %)	8 (20%)	3 (7.5 %)	11 (27.5 %)	1 (2.5 %)
	1	2	3	4	5	6	7	8	

Remarks (no categorisation)

- Unfortunately, I could only answer many questions with "I can't say" because the lunch meetings have been omitted. Overall, however, I think that it means an "improvement" in general.
- No information gain for me, data are known from conversations
- I checked "I can't say" since I don't feel that the results feed into our work at all, or I'm just not there if...?
- Had very few contacts with the study
- No contact with the study

**4. To what extent do you agree with the following statements?**	strongly disagree	somewhat disagree	somewhat agree	strongly agree	I can't say	Not answered
We succeeded in developing routines for the assessment and use of the questionnaire data that are suitable for everyday use.	3 (7.5 %)	10 (25 %)	9 (22.5 %)	5 (12.5 %)	12 (30 %)	1 (2.5 %)
The systematic/routine assessment of patient-reported distress helps to involve practitioners from different professions in the treatment in a more targeted way.	13 (32.5 %)	15 (37.5 %)	4 (10 %)	1 (2.5 %)	7 (17.5 %)	0 (0 %)
The systematic/routine assessment of patient-reported distress helps to better structure conversations and meetings.	7 (17.5 %)	15 (37.5 %)	8 (20 %)	1 (2.5 %)	8 (20 %)	1 (2.5 %)

5. The effort required to systematically/routinely assess patient-reported distress through the questionnaire is…

very low	rather low	rather high	very high	I can't say	Not answered
0 (0 %)	12 (30 %)	10 (25 %)	1 (2.5 %)	17 (42.5 %)	0 (0 %)

The benefit from the patient-reported questionnaire data in everyday work is…

very low	rather low	rather high	very high	I can`t say	Not answered
1 (2.5 %)	8 (20 %)	17 (42.5 %)	7 (17.5 %)	6 (15 %)	1 (2.5 %)

6. Generally, the patients' distress can be assessed through paper questionnaires or electronically. In your opinion, which of the two options is better suited to your institution?

O Paper questionnaires	7 (17.5 %)
O Electronic assessment	7 (17.5 %)
O Both options are sensible (paper questionnaires and electronic assessment)	19 (47.5 %)
O I cannot judge it	6 (15 %)
Not answered	1 (2.5 %)

7. Would you agree to continue the assessment of patient-reported distress through questionnaire?

Yes, without changes	Yes, with the following changes	No continuation, for the following reasons	Not answered
16 (40 %)	16 (40 %)	4 (10 %)	4 (10 %)

Remarks – original documentation, translated from German:

<u>Remarks of participants in favour of continuation:</u>

Means of assessment:
- Better embedding of data collection in the treatment process (e.g., during the initial interview)
- Electronic support via app
- It would be most useful if caregivers assessed the burden (third-party assessment and/or help with self-assessment)
- Patient fills out the questionnaire himself
- regular survey

Better integration of results:
- That it is discussed more in the team
- the information provided by the patients should be made more transparent for the whole team
- electronic acquisition + result must be visible in Meona [hospital information system software]. A field must be automatically created there, where the load and its change can be evaluated on a daily basis.
- Present results of the questionnaire in Meona or/and better incorporate into planning
- Implementation in Meona
- Involve nursing more in the process; discuss data more in the team.

Remarks on IPOS-questions
- simpler wording of the question on practical problems
- Electronic; strengthen patient self-reflection by offering visual scales of previously made statements!
- Additional question: What is the most important thing in the current situation? Wishes that do not concern the illness? What is helpful when things are bad?
- possibly other questions
- Topics --> the dying process, how to cope with it, how to cope with it for the relatives; allowing fears to be expressed and discussed intensively

Other:
- the benefit does not justify burdening the patient with filling it out in the palliative situation
- it would have to be an electronic collection, which is retrievable and evaluable. Someone would have to be in charge of the questionnaires - someone who takes care of it (evaluates which patient can do the IPOS; team discussion; assignment of tasks; control; filling out the IPOS together with the patient).
- lacks a person to do that

Remarks of participants against continuation:

- I often find it difficult to find the appropriate time for the patient, also I find that the results are not discussed enough, or I don't catch it
- additional effort for patient, little additional benefit for concrete work

8. What is your profession?	
O physician	12 (30 %)
O nurse	15 (37.5 %)
O psychosocial (psychology, social work, pastoral care, etc.)	9 (22.5 %)
O other	4 (10 %)

9. Where do you work?	
O palliative care ward	40 (100 %)
O specialised outpatient palliative care	0 (0 %)
O oncological day clinic or outpatient clinic	0 (0 %)

10. Further remarks (no categorisation)

- I will continue to use the questionnaire in the context of nursing rounds
- Overall a super idea! But the burdens must be recognizable for all. I.e. the information must reach everyone. Otherwise there is no added value. Unfortunately, I have "experienced" the latter.
- Communication in the team very good, interprofessional exchange takes place even without questionnaire. Therefore, despite regular integration, the questionnaire contributes little to the quality of care.
- When I looked at the completed questionnaires, I mainly paid attention to the area that concerns my tasks. I tended to skim over the other areas. Nevertheless, I had a good overall picture. In particular, the progressions were interesting for me, e.g. with regard to the assessment of whether problems have been addressed or which topics are still problematic and may need to be addressed again or what may have emerged.
- Stay healthy *heart*

Table S2: Exploratory correlations (Kendall's Tau) between items on use and items on benefit ratings /wish to continue PROM use

	**Benefit ratings**			**Wish to continue**
	notice of the patients' distress (Item 2)	useful for work (Item 1.7)	Treatment / support of patients improved (Item 1.8)	Wish to continue PROM use; dichotomous yes vs. no (Item 7)
**Indicators use**				
Reviewed questionnaire myself (Item 1.1)	.295*	.343*	.270	.021
Questionnaire discussed by team (Item 1.2)	.361**	.404**	.294**	-.141
Adapted treatment (Item 1.4)	.657**	.821**	.748**	-.461**
Addressed issues with patients (Item 1.5)	.587**	.653**	.740**	-.206
Addressed patients' burdens in team (Item 1.6)	.365**	.405**	.410**	.053

* p < .05 (two-sided)
** p < .01 (two-sided)

1 References

1. Etkind SN, Daveson BA, Kwok W, Witt J, Bausewein C, Higginson IJ, Murtagh FEM. Capture, transfer, and feedback of patient-centered outcomes data in palliative care populations: does it make a difference? A systematic review. Journal of pain and symptom management. 2015;49:611–24.
2. Antunes B, Harding R, Higginson IJ, EUROIMPACT. Implementing patient-reported outcome measures in palliative care clinical practice: a systematic review of facilitators and barriers. [Review]. Palliat Med. 2014;28:158–75. doi:10.1177/0269216313491619.
3. Bausewein C, Daveson BA, Currow DC, Downing J, Deliens L, Radbruch L, et al. EAPC White Paper on outcome measurement in palliative care: Improving practice, attaining outcomes and delivering quality services–Recommendations from the European Association for Palliative Care (EAPC) Task Force on Outcome Measurement. Palliat Med. 2016;30:6–22.
4. Bausewein C, Schildmann E, Rosenbruch J, Haberland B, Tänzler S, Ramsenthaler C. Starting from scratch: implementing outcome measurement in clinical practice. Ann Palliat Med. 2018;7:S253-S261. doi:10.21037/apm.2018.06.08.
