# Supplementary material for: Feasibility, use and benefits of patient-reported outcome measures in palliative care units: a multicentre observational study
Source: BMC Palliat Care. 2023 Jan 14;22:6. doi: 10.1186/s12904-022-01123-y (PMC9839955; doi:10.1186/s12904-022-01123-y)
Supplement: Supplementary file 2 — Additional file 2. Statistics on staff support for patients’ self-assessment of PROMs by type of discharge and point of measurement. [file 12904_2022_1123_MOESM2_ESM.pdf]

Additional file 2

Table S3: Statistics on staff support for patients' self-assessment of PROMs by type of discharge and point of measurement

	Patient groups based on reason for termination of data collection				
	**Regular discharge*** (n = 92)	**Death*** (n = 15)	**Deteriorated health*** (n = 21)	**Drop-out** (n = 9)	**Overall** (n = 137)
**Completion first measure point at admission: n (% column))**					
No support	29 (31.5)	3 (20.0)	3 (14.3)	1 (20.0)	36 (26.3)
Support	63 (68.5)	12 (80.0)	18 (85.7)	8 (80.0)	101 (73.7)
**Completion second measure point at 7 days or discharge: n (% column))**					
No support	20 (21.7)	2 (13.3)	1 (4.8)	0 (0.0)	23 (16.8)
Support	52 (56.5)	4 (26.7)	11 (23.8)	4 (44.4))	65 (47.4)
No second measurement	20 (21.8)	9 (60.0)	9 (71.4)	5 (55.6)	49 (35.8)

* For discharged patients, PROM assessment should have been carried out at discharge and therefore at least 2 measurement points were expected even if 7 days were not reached; for deceased patients or those with deteriorated health, one measure point was expected
