# Supplementary material for: Feasibility, use and benefits of patient-reported outcome measures in palliative care units: a multicentre observational study
Source: BMC Palliat Care. 2023 Jan 14;22:6. doi: 10.1186/s12904-022-01123-y (PMC9839955; doi:10.1186/s12904-022-01123-y)
Supplement: Supplementary file 3 — Additional file 3. Patient evaluation. [file 12904_2022_1123_MOESM3_ESM.pdf]

Additional File 3—Patient evaluation

<u>Methods</u>

Evaluation by patients: To ensure sufficient experience not just with completion but also with possible effects on communication with staff and treatment, the evaluation was conducted after completion of the second PROM or later. For evaluation, patients who had completed two or more IPOS questionnaires were visited by a study assistant and asked to complete the evaluation. Just like PROMs, completion was possible with or without support.

The questionnaire included only the most important aspects of feasibility from the study team's point of view. As the patient's health was likely to deteriorate during study participation in many cases, we hoped to ensure a higher participation rate by keeping the evaluation short.

Open-ended questions made it possible to comment on positive and negative experiences and study assistants actively asked patients to do so.

<u>Results of evaluation survey with patients (n (%))</u>

Sample: Of the 137 patients that completed IPOS at admission, 88 completed a second IPOS after one week / at discharge. Of these 88 patients, 73 completed evaluation. The reasons for non-participation in evaluation were not documented in all centres; however, in some cases, reasons were organisational (discharge before visit of the study assistant), and in others, patients did not want to participate in the evaluation.

The ***sample is highly selective***, which is important to know when interpreting the following data.

- Participants dying in inpatient care and patients whose health deteriorated during their stay dropped out of the data collection before evaluation. Thus, the results do not represent the views of patients in the last phase of life and those with strongly deteriorating health during their stay.

- What is more, patients that did not want to fill in questionnaires are likely to have refused participation and would not have been included in the sample either. Overall, 93 patients refused participation even though professionals assessed them as being able to complete PROMs. Patients were informed that the participation included PROM completion, a short evaluation and the collection of some socio-demographic and health data (mainly from routine documentation). However, they had to sign to allow the use of the data and we cannot know how many would have refused participation if the informed consent procedure and consent to data use had not been necessary (as would be the case in everyday practice).

Table S4: Questionnaire including results (n (%))

**Overall, how did you cope with the regular completion of the questionnaire?**					
very well	rather well	neither well nor badly	rather badly	badly	not answered
41 (56 %)	25 (34 %)	5 (4 %)	0 (0 %)	0 (0 %)	2 (3 %)
**Did you have the impression that your statements in the questionnaire were noticed and acted upon by your treatment team?** The treatment team includes all doctors, nurses and therapists involved in your treatment.					
always	often	sometimes	rarely	never	not answered
24 (33 %)	20 (27 %)	6 (8 %)	0 (0 %)	4 (5 %)	19 (26 %)
**Did you use your information in the questionnaires yourself and use it actively in conversations with the treatment team?**					
always	often	sometimes	rarely	never	not answered
10 (14 %)	22 (30 %)	12 (16 %)	3 (4 %)	22 (30 %)	4 (5 %)

How did you find the experience of reporting your burdens in the questionnaires?		
positive (e.g. informative / helpful / stimulating)	neutral	negative (e.g. boring / annoying / exhausting)
43 (59 %)	29 (40 %)	1 (1 %)
**Remarks sorted by category of patient's answer (positive / neutral /negative) – original documentation, translated from German:**		
**Situation of completion as positive experience**<br>• The conversation was very friendly. It was nice to talk to the study assistant about my stresses.<br>• I found the conversation with the staff member very positive. I am happy if I can help too.<br>• Because someone then had time to talk.<br>• The regular discussions did me a lot of good.<br>• Was important to be able to share<br>• Surprised how deep the questions go, I had to open up, but this helped me.<br>• Because questions were asked where the answers could be followed up.<br><br>**Reflection / feedback / assessment of one's own situation:**<br>• Partly helpful, some questions were thought-provoking.<br>• Because I had the feeling that people were talking about reality.<br>• Everything that came to light was good.<br>• I was able to reflect on my situation, which is also exhausting.<br>• Stimulating --> occupation, dealing with questions.<br>• Stimulating.<br>• It was purposeful. The problems addressed in the questionnaires are very important.<br><br>**Helpful – without further explanation:**<br>• Helpful.<br>• Helpful.<br><br>**Measure of quality improvement:**<br>• It is good that they try to improve the quality of the treatment in this way.<br><br>**Other:**<br>• I found the questions interesting.<br>• Unfortunately, I was often sleepy, which is why I was able to fill out the questionnaire less often than I had hoped.	**Remarks on IPOS-questions:**<br>• Much did not apply to me, hardly anything changed, therefore not useful for me.<br>• I think the intention behind the questionnaire is very good; the questions could be formulated more clearly.<br>• Mixture of multiple choice and answering 'free hand' unusual....<br><br>**Situation of completion / discussion of results as positive experience**<br>• I found the interview very pleasant.<br><br>**Doubt about benefit:**<br>• Difficult to judge. Whether it is really useful?<br><br>**Reflection / feedback / assessment of one's own situation:**<br>• Filling it out gave me a calm and neutral view of my stresses.<br><br>**Other:**<br>• Partly enlightening, partly annoying, exhausting.<br>• It didn't bother me too much - and since the survey is collected and evaluated nationwide - I told myself that I might do something good for the future.<br>• You also learn from it.	• Rather boring

You have regularly reported your stresses in the last few weeks and submitted them to your treatment team via a questionnaire.
Would you like to continue doing this (e.g. in contact with your specialist or family doctor)?

yes	rather yes	rather no	no	not answered
13 (18 %)	30 (41 %)	17 (23 %)	10 (14 %)	3 (4 %)

Reasons given by the patients in the open ended question – sorted by their answers (dichotomous)

Consideration on benefits of PROM use in later outpatient care:
- I would like to do the questionnaire with the family doctor. It is an important thing!
- So that the family doctor is informed
- I don't know if the GP would be able to evaluate the questionnaires, but he would also learn from the information.
- So you can tell the family doctor everything. Even without a questionnaire I tell everything, but with a questionnaire is better.
- Especially in my case that the general practitioner is up to date with my condition and can react as quickly as possible.
- I think it's good; you can say how you feel; otherwise you wouldn't do it.
- It's just a huge effort; whether the specialist/general practitioner would actually use the data is just the question; if so, I would take part; I also know about studies myself and support it. But all in all, the questionnaires are ok, you think more intensively about some questions.
- But: the GP has a lot to do, he would certainly not participate. If he would join in: rather yes.
- If you have already started, you can continue; would then be in the hospice
- It is always good to have a continuous exchange.

Possible personal / additional / future benefit:
- If I can improve my situation, why not?
- To continue to improve the situation --> also for other patients.
- Because it probably brings something.
- I see advantages in this towards me. The more information the treatment team has, the better they can react.
- Health condition further developed through much consultation / communication = important

Reflection / feedback / assessment of one's own situation:
- Reflection, feedback, concentration on the essentials can't hurt :)
- It can help me to better assess my situation.
- Structured procedure - otherwise you might forget things.
- Because the questions are relevant to me
- You become aware once again of where you stand and what building sites are currently there!

Other:
- Somehow the questions always seem different to me; oh, they are always the same questions?
- Because I find it interesting how carefully the questions are posed, so that you are not nailed down to a certain answer.
- No reason not to continue.
- Why not, I have nothing to hide?
- Why should you end something positive?
- it does no harm
- Because I think my illness will drag on.

Consideration on benefits of PROM use in outpatient care:
- 'They have NO adequate staff (and certainly not for something like this) and are already stressed today and have NO TIME.'
- Implementation here on the ward is guaranteed (interest on the part of the doctors); in a nursing home, however, there is no time for this.
- For the above reasons; my family doctor would thank me if I arrived with so many slips of paper.
- Because I would no longer get any feedback (the treatment team is no longer responsible for me ...).
- Bad contact with doctor.

No personal / additional benefit:
- I didn't need it before and I don't see any added value in it for me.
- I don't need questionnaires during my treatment. The conversation is enough.
- Because it doesn't help me personally. I would have got more out of resting than answering the questions and telling the same thing over and over again.
- More out of clinic interest, a little for me.
- My family doctor is my friend, so there is close contact.

Too exhausting / need of support:
- I would need help with reading aloud and writing.
- exhausting
- Filling out the questionnaire - even with help - is too strenuous for me.
- Because I have already answered so many questions and had so many conversations, which also takes a lot of energy

Not necessary:
- A continuation of the questionnaires is not absolutely necessary.
- I do not necessarily need this any further.

Other:
- I don't really want to continue with the questionnaires.
- Overall, I don't like questionnaires very much. I answered these questionnaires because you were so nice.
- Because it was boring
- Time problems
  Not possible in the near future

- I do not know
- As long as the condition allows it.
